# Supplementary material for: Amino Acid Absorption Profiles in Growing Pigs Fed Different Protein Sources
Source: Animals (Basel). 2021 Jun 10;11(6):1740. doi: 10.3390/ani11061740 (PMC8246322; doi:10.3390/ani11061740)
Supplement: Supplementary file 1 [file animals-11-01740-s001.zip › animals-1235714-supplementary.pdf]

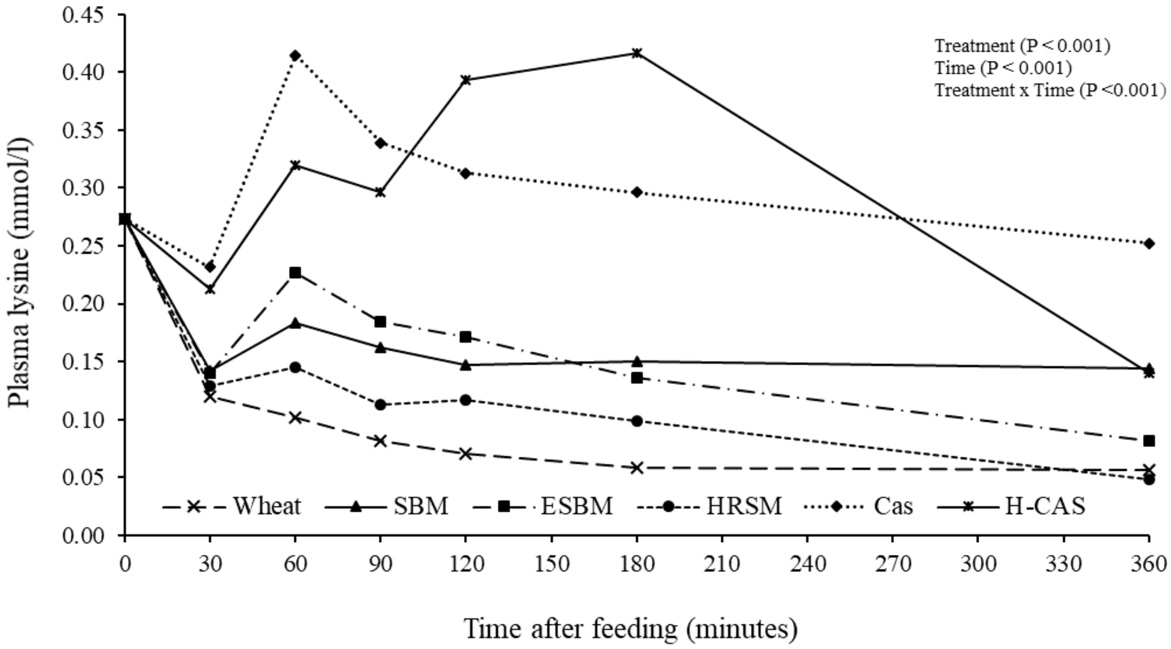

Figure S1. Postprandial lysine (Lys) concentration in plasma of growing pigs fed experimental diets containing wheat, soybean meal (SBM), enzyme-treated soybean meal (ESBM), hydrothermally treated rapeseed meal (HRSM), casein (Cas) and hydrolysed casein (H-Cas) as the only dietary protein sources. Values are least squares means with five replicates.

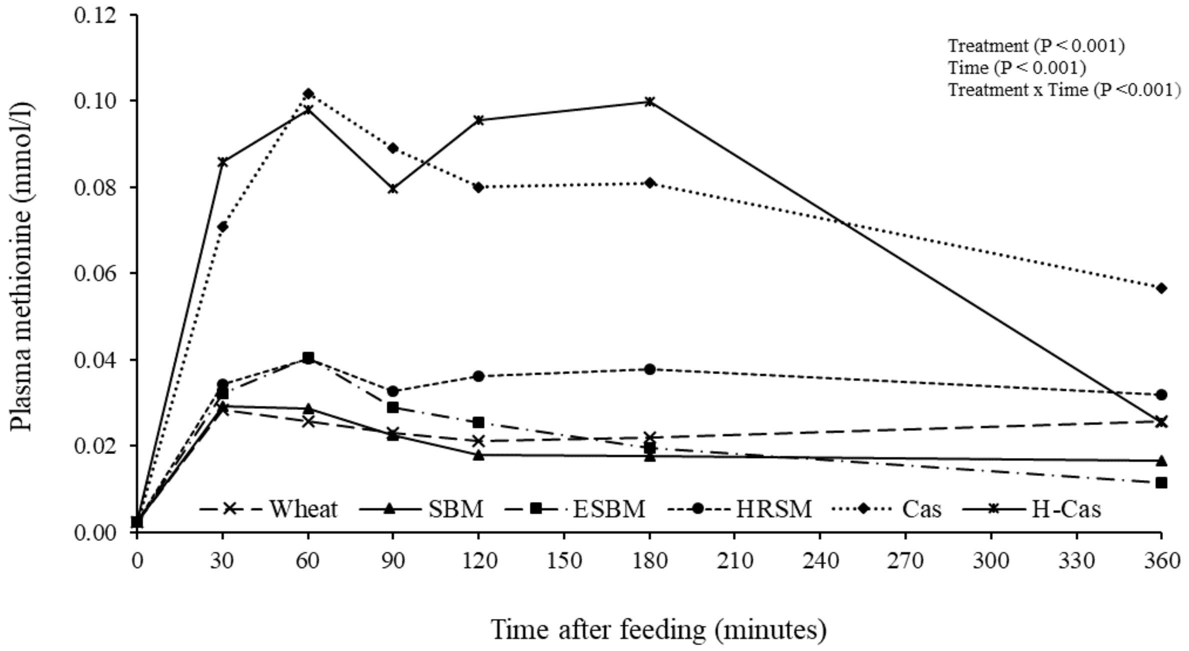

Figure S2. Postprandial methionine (Met) concentration in plasma of growing pigs fed experimental diets containing wheat, soybean meal (SBM), enzyme-treated soybean meal (ESBM), hydrothermally treated rapeseed meal (HRSM), casein (Cas) and hydrolysed casein (H-Cas) as the only dietary protein sources. Values are least squares means with five replicates.
